# Editorials

**Published:** 1908-11

**Authors:** 


### EDITORIALS

The Business Office of the JOURNAL-RECORD is 1 1-2 to 5 1-2 South Broad St.
The Editorial Office is 1014-15 Century Bldg.
Address all Business Communications to Journal-Record of Medicine, 1 1-2 to 5 1-2 South Broad Street.
Make remittances payable to THE JOURNAL-RECORD OF MEDICINE.
On matters pertaining to the Editorial and Original communications, address Edgar G. Ballenger, M. D., Atlanta, Ga.
Reprints of original articles will be furnished at cost price. Requests for the same should always be made in THE MANUSCRIPT.
We will present, postpaid, on request, to each contributor of an original article, twenty (20) marked copies of THE JOURNAL-RECORD OF MEDICINE containing such article.

### THE INTERNATIONAL CONGRESS ON TUBERCULOSIS

The Sixth International Congress on Tuberculosis, which met in Washington, D. C., from September 28th to October 3rd, was a marked success, of which every American, and especially the members of the medical profession may justly feel proud. It was a success in every way, from attendance and scientific papers to the weather and entertainments. The exhibits were the best ever presented to the public, of great variety, interesting and instructive, and of much educational value, both to the profession and laity. They opened September 21, and continued until October 12th.

The section meeting which was really the congress, began with a general meeting September 28th, and closed with another general meeting October 3rd.

Over these general meetings, Secretary Cortelyou presided as the special representative of President Roosevelt. At each of these meetings, Dr. John S. Fulton, the secretary general, called the roster of the nations and over a score of foreign governments responded through their official representatives. The oldest and the youngest nations were there, China and Panama. The largest and smallest, Russia and Belgium, thus manifesting the world wide interest taken in the study of the Great White Plague. Every state in the union was also represented by delegations varying in size from three to a hundred. Georgia had about a dozen present.

One could not fail to be impressed with the linguistic accomplishments of the foreign physicians, for while French, German and Spanish were the official languages of the Congress, as well as English, nearly all spoke the latter accurately and fluently. This was particularly noticable with the Japs and Chinese.

At the last general session President Roosevelt made a splendid address to the congress in which he gave an appreciative review of the noble work done by the medical profession in stamping out disease, and alluded especially to what had been accomplished by the president of the American Medical Association, Col. Gorgas, in Panama.

Prof. Robert Koch was there, of course, for a Tuberculosis Congress without Koch, would be like the play of Hamlet with Hamlet left out. Naturally he was the cynosure of all eyes when he appeared, but his pet theory on the non-transmissibility of bovine tuberculosis to man received a pretty severe jolt.

At a joint meeting of sections 1 and 7, (Pathology and Bacteriology and Tuberculosis in Animals) Prof. Koch reiterated the statement made at the London Congress in 1905, but Prof. Woodhead, of Cambridge, England; Prof. Arloying, of Lyons, France, Prof. Ravenel, of University of Wisconsin, and Prof. Tendeloo, of Leyden, and others, took issue with him, and seemed to have the facts and figures to prove the transmissibility of bovine tuberculosis to man. A private conference was held in which quite a number of the highest authorities tried to get Prof. Koch to modify his position, but he declined, and the discussion waxed quite warm at times. Had it not been for the respect all felt for him, and personal considerations, a resolution condemning it would have been passed.

The scientific work of the Congress was carried on in seven sections:

Sec. 1.—*Pathology and Bacteriology.*
Pres. Dr. Wm. H. Welch, Baltimore.

Sec. 2.—*Sanatoria, Hospitals and Dispensaries.*
Pres. Dr. Vincent Y. Bowditch, Boston.

Sec. 3.—*Surgery and Orthepedics.*
Pres. Dr. Chas. H. Mayo, Rochester, Minn.

Sec. 4.—*Tuberculosis in Children.*
Pres. Dr. Abram Jacobi, New York.

Sec. 5.—*Hygiene, Social, Industrial and Economic Aspects of Tuberculosis.*

Pres. Mr. Edward T. Devine, New York.

Sec. 6.—*State and Municipal Control of Tuberculosis.*

Pres. Surg.-Genl. Walter Wyman, Washington, D. C.

Sec. 7.—*Tuberculosis in Animals and Its Relation to Man.*

Pres. Dr. Leonard Pearson, Philadelphia.

Of course, it will be impossible even to give a brief summary of the many valuable papers presented in the different sections, for they were all going on at the same time, and one will have to bide the time with patience until the proceedings are published, when one can "read, mark, learn, and inwardly digest" these valuable contributions to the literature on tuberculosis.

The value of tuberculin, not only as a diagnostic agent, but as a curative agent, was generally acknowledged by all, and it is used at Saranac Lake and in nearly all the sanatoria, both in Europe and America. As to the best method of using for diagnostic purposes, whether hypodermically, conjunctival or integumental, there was naturally a difference of opinion. This same difference existed as to amount of reaction and dose to be administered when using as a theraputic agent, but all showed that progress was being made in treatment. All agreed on the importance of living in the open air, diet, and rest, which can best be had at sanatoria, but can be carried out at home. A change of climate was not considered necessary for the successful treatment of tuberculosis, though it is beneficial in some cases.

The committee on resolutions presented the following, which were adopted as expressed of the sentiment of the Congress.

First.— The attention of state and central governments should be called to the importance of proper laws for the obligatory notification of medical attendants to proper health authorities, of all cases of tuberculosis, coming to their notice, and for the registration of such cases, in order to enable the health authorities to put into operation adequate measures for the prevention of the disease. We urge on the public and all governments the establishment of hospitals for the treatment of advanced cases of tuberculosis.

Second.—We urge the establishment of sanatoria for curable cases of tuberculosis.

Third.—We urge the establishment of dispensaries and day camps, and camps for ambulant cases of tuberculosis which cannot enter hospitals and sanatoria. Again the utmost efforts should be continued in the struggle against tuberculosis, to prevent conveyance from man to man of tuberculous infection, as the most important source of the disease. Further preventive measures must be continued against bovine tuberculosis, and the possibility of the propagation of this to man should be recognized.

Resolved, That this Congress endorses such well considered legislation for the regulation of factories and workshops, the abolition of premature and injurious labor to women and children, and the securing of sanitary dwellings as will increase the resisting power of the individual to tuberculosis and other diseases; that instruction in personal and school hygiene should be given in all schools for the professional training of teachers; that wherever possible such instruction in elementary hygiene should be instructed to properly qualified medical instructors; that colleges and universities should be urged to establish courses in hygiene and sanitation, and also include these subjects among their entrance requirements to stimulate more useful elementary instructions in the lower schools. That this Congress endorses the establishment of play grounds as an important means to preventing tuberculosis through their influence on health and resistance to disease.

A spirit of optimism prevaded the Congress, and its members believed that in time tuberculosis will be under as complete control as small-pox is today. As to when that happy time will arrive, there was quite a diversity of opinion, some of the most sanguine placing it only at thirty years, while the conservatives expected this happy consummation as the crowning victory of their warfare on the Great White Plague by the close of the 20th Century. To accomplish this the medical profession must have the co-operation of the laity, and it was one of the hopeful signs of the progress made, to see the interest manifested in this Congress by such philanthropists as Mr. Henry Phipps, of New York, who has given over a million dollars to aid in the fight on tuberculosis.

The social features of the Congress were very pleasant, and the arrangements and entertainments were all that could have been desired; and those having these in charge have the congratulations and thanks of all who had the pleasure of attending.

H. R. S.

(It is with pleasure that we are enabled to publish the above review of the recent Congress on Tuberculosis by Dr. H. R. Slack, of LaGrange, who has kindly complied with the editor's request and presented the personal views of a Georgian who is much interested in and thoroughly conversant with tuberculosis and its many different phases).

---

### SOUTHERN MEDICAL ASSOCIATION.

As we go to press the second annual meeting of the Southern Medical Association closes after a most successful meeting. The large attendence, the excellence of the majority of the papers presented by the members in promoting the welfare of the Association clearly indicated continued success and a rapid growth for this body of medical men, which should number among its members every man in our Southland who has any interest in the scientific advancement of the medical profession.

The officers of the association deserve great credit for the success of this meetting, but especial mention should be made of the untiring efforts of Dr. B. L. Wyman, our president; our courteous and able secretary-treasurer, Dr. Oscar Dowling, Shreveport, La., without whose careful attention we could not have heard so many papers by well known Southern physicians. It is with much pleasure that we note the re-election of Dr. Dowling to this position to which he is so well adapted, both by his ability and by his energy in looking out for the interests of the association.

We believe that the time limit for papers should be reduced from 20 to 10 minutes and that, with this reduction and a strict enforcement of the 5 minute limit for discussions, the entire association might meet in a single body where all the papers might be read before the entire association instead of a section. All papers might be condensed so as to be read in 5 or 10 minutes and the additional interest that would follow the reading of short crisp papers would more than justify such a change. This plan would provide a larger audience for all contributors and would tend to educate the members in subjects outside of the fields in which their own individual efforts confine them in looking out for the best interests of the association.

New Orleans was selected as the next meeting place and the following officers were elected for the ensuing year:

President, Dr. Giles C. Savage, of Nashville, Tenn.; vice-presidents (one from each state): J. M. Jackson, Jr., of Miami, for Florida; Charles M. Murry, of Ripley, for Mississippi; George Dock, of New Orleans, for Louisiana; T. A. Casey, of Birmingham, for Alabama; J. C. Olmstead, of Atlanta, for Georgia, and E. C. Ellett, of Memphis, for Tennessee.

Dr. Oscar Dowling, of Shreveport, La., was re-elected to the office of secretary.

After his induction into office, Dr. Savage, the new president, announced the appointment of the following new councilors for the full three-year term: Dr. W. W. Crawford, of Mississippi, to succeed himself; Dr. B. L. Wyman, of Birmingham, to succeed Dr. D. F. Talley, of Birmingham, and Dr. West, of Chattanooga, to succeed Dr. Savage. The three other councilors are Michael Hoke, of Atlanta; John McDiarmid, of DeLand, Fla., and W. W. Butterworth, of New Orleans. Two of the last named three have one year yet to serve, and the third has two years' service remaining.

The following section officers were elected by the three sections and their names were announced to the general session: Dr. F. G. DuBose, of Mobile, Ala., chairman, and Dr. Jere L. Crook, of Jackson, Tenn., secretary of the section on surgery; Dr. J. A. Witherspoon, of Nashville, Tenn., chairman, and Dr. H. L. Mitchell, of Birmingham, secretary, of the section on medicines, and Dr. A. W. Stirling, of Atlanta, chairman and Dr. A. B. Harris, of Birmingham, secretary, of the section on opthalmology.

The courtesies extended by the New Kimball were highly appreciated by all connected with the association.

---

Dr. De Forest Willard, professor of orthopaedic surgery at the University of Pennsylvania, has been appointed consulting orthopaedic surgeon at the Municipal Hospital, Philadelphia.

